# The Adulterated Food Law in Texas a Dead Letter

**Published:** 1899-06

**Authors:** 


### The Adulterated Food Law in Texas a Dead Letter.

It is not generally known, even to the doctors and the law-makers of Texas, that there is such a law on the statute books of Texas as the following; the law-makers of subsequent assemblies I suppose never heard of it. It has never been executed, although passed in 1883. It is to be found under Title 91 in the Revised Statutes of Texas, 1895, page 865, and Articles 4317,4318,4319 and 4320; and also under those figures in Sayles' Digest, 1897, as stated by the Committee on State Board of Health of the Texas State Medical Association in their memorial to the Governor (published in this journal for May, ult., and reprints of which have been sent to the members of the Legislature and to the Governor.)

Section 1 reads as follows:

"State Health Officer—Powers and Duty of.—The State Health Officer shall take cognizance of the interests of the public health, as it relates to the sale of food and drugs, and the adulteration of the same, and make all necessary investigations and inquiries relating thereto. He shall also have the supervision of the appointment of public analysts and chemists, and upon his recommendation, whenever he shall deem any such officer incompetent, the appointment of any and every such officer shall be revoked, and be held to be void and of no effect. Within thirty days after the passage of this act, the State Health Officer shall adopt such measures as may seem necessary to facilitate the enforcement of this act, and prepare rules and regulations with regard to the proper method of collecting and examining articles of food or drugs, and for the appointment of the necessary inspectors and analysts, and the said health officer shall be authorized to expend an amount not exceeding two thousand dollars, for the purpose of carrying out the provisions of this act."

(The other articles and sections relate to details, as follows: "Sale of adulterated food, drinks and drugs, prohibited: food; drug; defined. Adulteration of drugs, food and drinks, defined. Exempt articles; variability from standard defined. Samples of foods or drugs (to be) furnished for analysis. Violation of act (how) punished. Repealing clause. Regulations of State Health Officer (to be) printed," etc.)

It is due to the State Health Officer and to the memory of his predecessor to say that it is no fault of theirs that the law has never been executed. There has never been any appropriation made for carrying it into effect. The writer served in the State Health Office nearly eight years, and to his knowledge, there was never any appropriation made, except for strictly quarantine purposes and the necessary buildings and accessories, and that was always meagre and often insufficient. Moreover, each item was specified for which the money was to be used. Were the two thousand dollars, which the law says the State Health Officer "is authorized to expend in carrying out this act," made available each session of the Legislature (every two years), it would be totally inadequate to the purposes. How many "analysts, chemists and inspectors" would it pay for two years? to say nothing of any other expense. Besides this, the law is fundamentally and essentially defective, and would be inoperative, even were sufficient funds available; for instance: The law says "the State Health Officer shall have the *supervision* of the appointment of analysts and chemists," etc., but does not say how, nor by whom they shall be appointed; nor how many there shall be; nor how much, nor by whom they shall be paid, etc. Nor does the act provide for paying them, nor for any clerical assistance in the office of the State Health Officer; nor for the vast amount of printing that he is required to have done; nor for postage, etc., yet it would require of him duties that necessitate the employment of a considerable force of clerks and correspondents. The law is crude and ill considered, and is a disgrace to the statute books; it should be omitted in the next revision. As niggardly as have been all the Texas Legislatures in the matter of paying for clerks in the several departments, they have never allowed the State Health Officer any office assistance whatever, except one secretary at starvation wages. I can bear testimony to the fact, as will the records, that the late State Health Officer several times asked for the appropriation of $2,000 a year,—not for this fool law, but to employ a chemist and bacteriologist to make investigations into the causes of certain local epidemics, and it was always refused. On one occasion when he was before the august Committee on Public Health, and they were screwing him down so closely in the matter of quarantine expenses, he became indignant, and rising from his chair, said: "Gentlemen, do not suppose for an instant that it matters with me, personally, what amount you appropriate, or whether you *make any appropriation at all.* If you can afford to let the quarantine lapse into total neglect, I can. Good morning."

The above has been written after looking into the matter. In the "Memorial," and in the report of the Committee on State Board of Health, this law was cited, and the gentlemen seem to think that there is no reason why it should not be enforced, and apparently attach blame to the State Health Officer, present and past, for its neglect. I thought at first that it might be resurrected, and if the matter of an appropriation of $2,000 was all that was in the way, that might be remedied, perhaps; but upon examination of the act I feel assured that it is past praying for.

